# Dose-dependent severe cutaneous reactions to imatinib

**DOI:** 10.1038/sj.bjc.6600893

**Published:** 2003-04-15

**Authors:** S Ugurel, R Hildenbrand, E Dippel, A Hochhaus, D Schadendorf

**Affiliations:** 1 Skin Cancer Unit, German Cancer Research Center, 69120 Heidelberg, Germany; 2 Institute of Pathology, University Hospital of Mannheim, 68167 Mannheim, Germany; 3 Department of Dermatology, University Hospital of Mannheim, 68167 Mannheim, Germany; 4 Department of Internal Medicine, University Hospital of Mannheim, 68167 Mannheim, Germany

**Keywords:** imatinib, melanoma, adverse drug reaction, c-kit, mast cells

## Abstract

The protein kinase inhibitor imatinib has been approved as an efficient anticancer drug with common but mild cutaneous toxicities. We here report on two out of four melanoma patients treated with high-dose imatinib presenting with severe and strongly dose-dependent skin eruptions, suggesting a cutaneous reactivity pattern different from allergic hypersensitivity.

Imatinib mesylate (Gleevec, formerly STI571, Novartis, Basel, Switzerland) is a selective inhibitor of the tyrosine kinase family including the oncogenes Abl and Kit as well as the platelet-derived growth factor receptor (PDGF-R). Owing to its mechanisms of action, imatinib has been used successfully in chronic myelogenous leukaemia (CML) and in gastrointestinal stromal tumours (GIST). Dose-escalation studies proved imatinib as a well-tolerated oral drug with rare dose-limiting toxicities (Druker *et al*, 2001). Nevertheless, severe cutaneous side effects have been reported, leading to a cessation of imatinib treatment in single cases (Brouard and Saurat, 2001).

We here report on the first four patients being enrolled into a clinical phase II study investigating imatinib as a second-line therapy in advanced melanoma. The study's rationale was based on the well-known expression of c-kit as well as PDGF-R in melanoma cell lines and tissues (Ohashi *et al*, 1996). Albeit the patients' clinical outcome still has to be awaited, we hereby provide a first report of the noticeable strong and frequent cutaneous adverse events.

## DESCRIPTION OF CASES

All four patients received imatinib 400 mg b.i.d. (800 mg day^−1^) after progression of melanoma disease under a first-line cytostatic regimen was ascertained. Patients' clinical data are summarized in Table 1(A)

**Table 1:** Skin reactions to imatinib

**(A)**	**Age (years)/gender**	**Localization of metastases**	**Skin reaction**	**CTC grading**	**Onset of reaction (days after first application of imatinib)**	**Dose adjustment**
Patient 1	71/m	Liver, spleen, subcutaneous	None	0	None	None
Patient 2	51/f	Lung, subcutaneous	Generalised macular and urticarial eruption	3	10	50% reduction
Patient 3	41/f	Lymph nodes, subcutaneous	Generalized macular and urticarial eruption	3	5	50% reduction, corticosteroids
Patient 4	69/f	Skin	Macular rash of the face	2	7	None
**(B)**	**Author, year of publication**	**Imatinib dose (mg day^−1^)**	**Number of patients treated**		**Mild or moderate cutaneous reactions (CTC grade 1–2)**	**Severe cutaneous reactions (CTC grade 3–4)**
CML	Kantarjian *et al* (2002)	400	532		155 (29.1%)	16 (3.0%)
CML	Talpaz *et al* (2002)	400–600	235		49 (20.8%)	3 (1.3%)
CML	Sawyers *et al* (2002)	400–600	260		48 (18.5%)	11 (4.2%)
GIST	van Oosterom *et al* (2001)	400–1000	40		17 (42.5%)	5 (12.5%)
GIST	Demetri *et al* (2002)	400–600	147		41 (27.9%)	4 (2.7%)

(A) Melanoma patients treated with imatinib 800 mg day^−1^. (B) CML and GIST patients treated with different doses of imatinib. CTC=common toxicity criteria of the National Cancer Institute, National Institutes of Health; CML=chronic myelogenous leukaemia; GIST=gastrointestinal stromal tumour.

**Figure 1:**
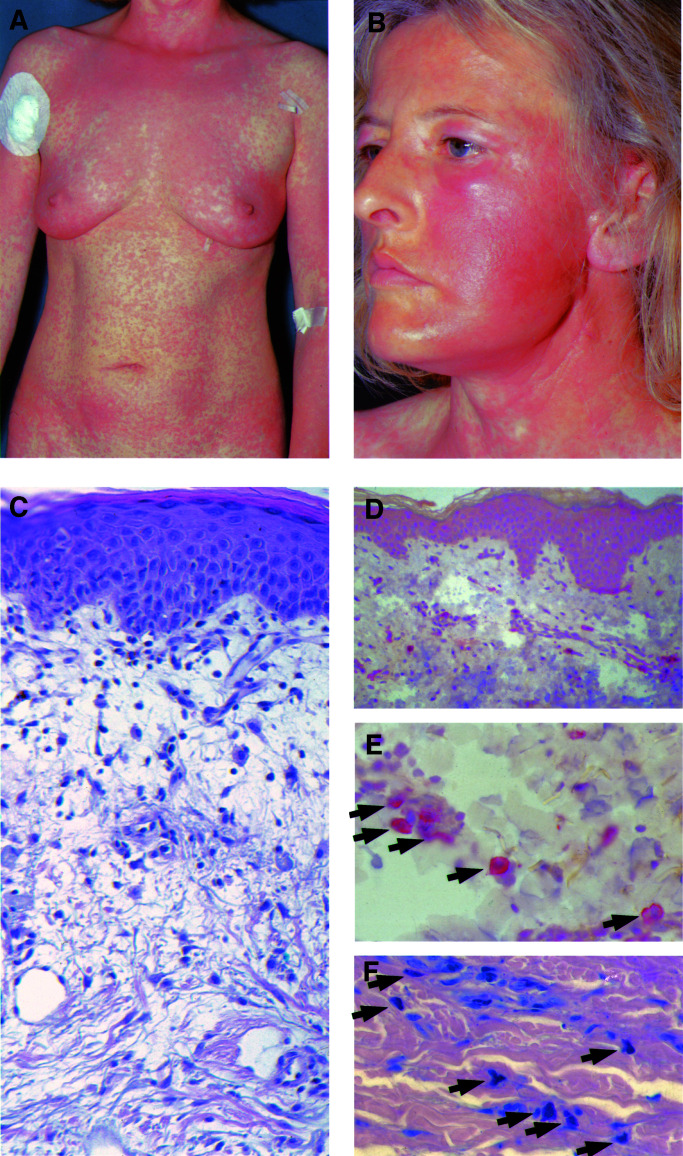
Photographs and photomicrographs of patient 3 at day 10 after the onset of treatment with imatinib 800 mg day^−1^. (**A**, **B**) Face and trunk of the patient showing generalised macular and urticarial cutaneous eruptions. (**C**) Haematoxylin–eosin staining of lesional skin revealing a mononuclear infiltrate and marked oedema of the upper dermis; magnification 1 : 200. (**D**, **E**) Immunohistochemical staining of lesional skin showing strong expression of c-kit (CD117) in discrete mononuclear cells (arrows) of the upper dermis; magnification 1 : 100 (D) and 1 : 400 (**E**). (**F**) Giemsa staining revealing an increased number of mast cells (arrows) in the upper dermis of lesional skin; magnification 1 : 400.

. Three of four patients showed moderate to strong cutaneous reactions with a macular and/or urticarial pattern within the first two weeks of therapy (Figure 1A, B

). The exanthema emanated from the face and trunk, and subsequently afflicted the extremities. Other side effects were of mild to moderate intensity including fluid retention, nausea, vomiting and fatigue. As a result of the severity of skin reactions, 50% dose adjustments became necessary in two of three patients. Additionally, corticosteroids (methylprednisolon 60 mg day^−1^ p.o.) were given in one patient. The cutaneous eruptions regressed in all three patients within 2–3 weeks. Thereafter, imatinib was again escalated to 75% of the initial dose (600 mg day^−1^) in patients 2 and 3, leading to a strong relapse of skin eruptions in both cases. During the further course of treatment, imatinib was left at 50% reduction (400 mg day^−1^) in these two patients, resulting in no further skin reactions.

## HISTOLOGICAL ANALYSES AND DISCUSSION

A review of the literature revealed mild to moderate cutaneous reactions as a common side effect to imatinib (Table 1(B)). In contrast, severe skin reactions are rarely reported but, however, occurred following high treatment doses of 600–1000 mg day^−1^ as used in a phase I trial in GIST (van Oosterom *et al*, 2001) as well as in our ongoing study. Histological analyses of biopsy specimens of lesional skin from patients 2 and 3 revealed loose mononuclear cells infiltrating the dermis with a perivascular pattern (Figure 1C). The upper dermis showed marked oedema and, remarkably, an increased number of mast cells. Immunohistochemical analyses using polyclonal goat-anti-human antibodies A4502 (DAKO, Hamburg, Germany) recognising the c-kit gene product CD117 revealed a strong staining of mononuclear cells of the dermis morphologically corresponding to mast cells (Figure 1D, E), as additionally confirmed by Giemsa staining (Figure 1F). Human mast cells, which express a functional c-kit receptor, are known to be susceptible to tyrosine kinase inhibition by imatinib leading to impaired proliferation as well as induction of apoptosis (Ma *et al*, 2002). Therefore, an enhanced proliferation of mast cells as observed in our patients seems to be unlikely mediated via c-kit inhibition on the mast cells themselves. We therefore suggest imatinib to act as a dose-dependent inducer of chemoattractant substances like, for example, cytokines and growth factors leading to dermal mast cell accumulation. Nevertheless, the precise mechanism of action needs to be further elucidated.

We conclude that imatinib acts as a dose-dependent inducer of cutaneous adverse reactions with mainly mild reactivity to low or intermediate doses (200–600 mg day^−1^) but severe eruptions to high doses (600–1000 mg day^−1^) of the drug. Our findings should stimulate further investigation of the cutaneous reaction profile to imatinib. To avoid strong cutaneous adverse reactions, however, treatment with low or intermediate doses of imatinib should be preferred in comparison with high doses of this drug whenever feasible.
